# Role of anatomical location, cellular phenotype and perfusion of adipose tissue in intermediary metabolism: A narrative review

**DOI:** 10.1007/s11154-021-09708-3

**Published:** 2022-01-15

**Authors:** Stefania Camastra, Ele Ferrannini

**Affiliations:** 1 Department of Clinical & Experimental Medicine, University of Pisa, Pisa, Italy; 2 Institute of Clinical Physiology, CNR, Pisa, Italy

**Keywords:** Adipose tissue, Adipocytes, Insulin sensitivity, Blood flow, FFA, Glucose metabolism

## Abstract

It is well-established that adipose tissue accumulation is associated with insulin resistance through multiple mechanisms. One major metabolic link is the classical Randle cycle: enhanced release of free fatty acids (FFA) from hydrolysis of adipose tissue triglycerides impedes insulin-mediated glucose uptake in muscle tissues. Less well studied are the different routes of this communication. First, white adipose tissue depots may be regionally distant from muscle (*i.e.,* gluteal fat and diaphragm muscle) or contiguous to muscle but separated by a fascia (Scarpa’s *fascia* in the abdomen, *fascia lata* in the thigh). In this case, released FFA outflow through the venous drainage and merge into arterial plasma to be transported to muscle tissues. Next, cytosolic triglycerides can directly, *i.e.,* within the cell, provide FFA to myocytes (but also pancreatic ß-cells, renal tubular cells, etc*.*). Finally, adipocyte layers or lumps may be adjacent to, but not anatomically segregated, from muscle, as is typically the case for epicardial fat and cardiomyocytes. As regulation of these three main delivery paths is different, their separate contribution to substrate competition at the whole-body level is uncertain. Another important link between fat and muscle is vascular. In the resting state, blood flow is generally higher in adipose tissue than in muscle. In the insulinized state, fat blood flow is directly related to whole-body insulin resistance whereas muscle blood flow is not; consequently, fractional (*i.e.,* flow-adjusted) glucose uptake is stimulated in muscle but not fat. Thus, reduced blood supply is a major factor for the impairment of *in vivo* insulin-mediated glucose uptake in both subcutaneous and visceral fat. In contrast, the insulin resistance of glucose uptake in resting skeletal muscle is predominantly a cellular defect.

## Introduction

Previously regarded as an inert depot for storage of energy-rich substrates, in the past two decades adipose tissue (AT) has taken up an important role as a major signaling, metabolic, biomechanical, and innate immunity organ both in normal physiology and disease [1]. With the advance of imaging techniques, it has been possible to quantitate fat depots *in vivo* in organs such as muscle, liver, pancreas, and heart, and their relationship with neighboring tissues [2–7]. In addition, important details about the cellular phenotype of adipocytes in different sites have been gained using refined histology on human AT biopsy specimens. Finally, physiological studies have provided insight into the role of perfusion in the response of adipose tissue to insulin. Here, we focus on these aspects in particular as they relate to glucose metabolism.

## FFA and glucose: anatomical factors

Free fatty acids (FFA) and glucose are main substrates for virtually all bodily tissues. Their relative use for energy production is regulated in a biochemically reciprocal fashion, epitomized as the Randle cycle. The overall contribution of AT to glucose metabolism depends on its mass, location and vascularization [5, 8–12].

**Fig. 1:**
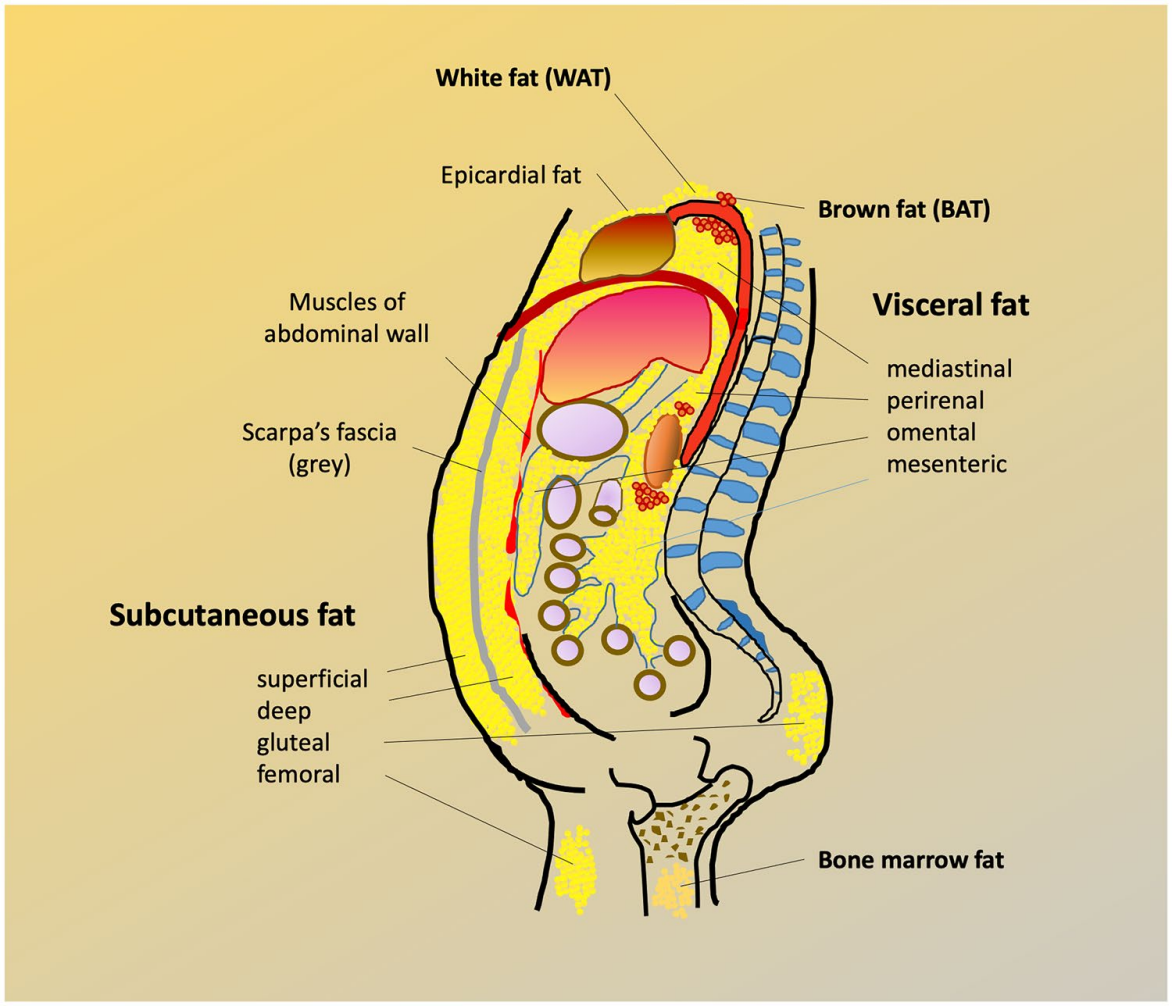
Sketch of the main human adipose tissue depots. WAT = white adipose tissue; BAT = brown adipose tissue

**Fig. 2:**
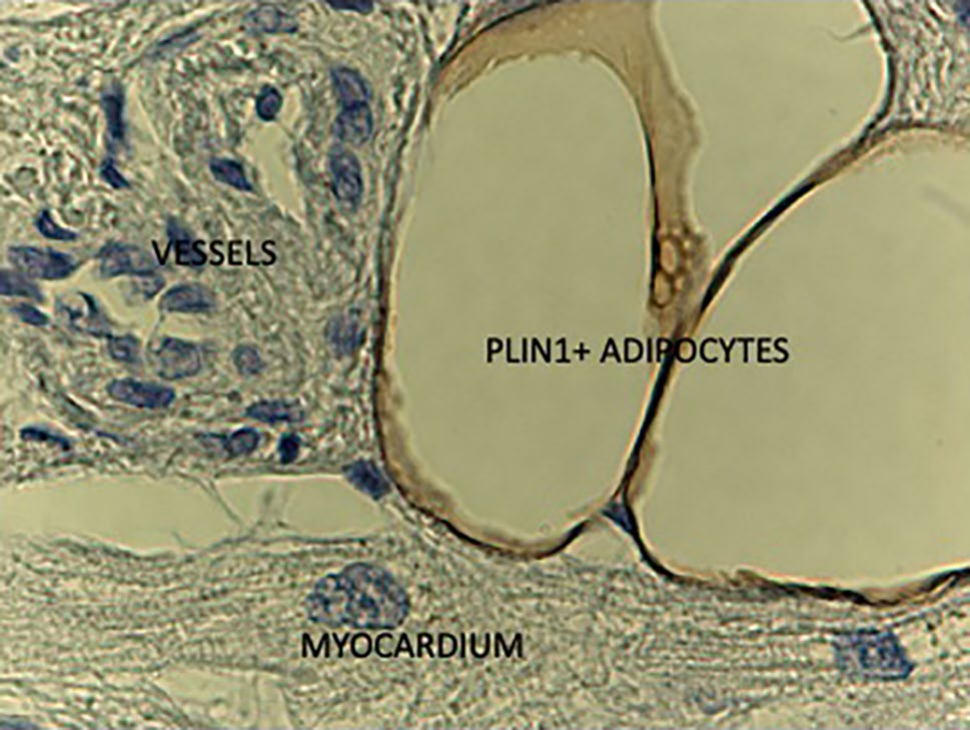
Human atrial myocardium biopsy: perilipin1 (PLIN1) immunoreactive adipocytes, myocardial muscle fiber and pre-capillary vessels. (Courtesy of S. Cinti UnivPM Ancona, Italy)

- AT accumulates predominantly as subcutaneous fat (SAT) [8], and, to a lesser extent, as visceral fat (VAT), which in humans includes intraperitoneal (omental, mesenteric), retroperitoneal, mediastinal, gonadal, and pericardial AT [13]. Though a minor fraction of whole body fat, VAT expansion is associated with more severe metabolic and cardiovascular damage than SAT [14].
- SAT venous efflux is a tributary of the systemic venous return, so that FFA outflow from SAT is diluted into the general circulation. In contrast, abdominal VAT drains through the portal route, thereby exposing the liver to higher FFA delivery than the hepatic artery. Given the small size of VAT *vs* SAT, FFA originating from abdominal VAT only make up for ~ 20% of arterial FFA concentration [15].
- White adipose depots may be regionally distant from other organs (*e.g.,* gluteal fat from diaphragm muscle) or contiguous to another organ but separated by a fascia (*e.g., fascia lata* in the thigh, renal capsule and perirenal fat) (Fig. 1). In this case, FFA leave AT through the venous drainage and merge into arterial plasma to be transported to tissues; collectively, this AT constitutes a proper AT ‘organ’.
- Cytosolic triglycerides can directly, *i.e.,* within the cell, provide FFA to myocytes (but also hepatocytes, renal tubular cells, etc*.*) [16, 17]. In this case, triglyceride hydrolysis presumably contributes little to the arterial FFA pool, although this route is difficult to quantify *in vivo*.
- An intermediate situation is the presence of adipocyte layers adjacent to, but not anatomically segregated, from muscle. An example of this arrangement is epicardial fat overlaying the myocardium. Yet another arrangement is the presence of adipocyte clumps interspersed within another tissue, *e.g.,* the myocardium [18]. As exemplified in Fig. 2, adipocytes are wedged between precapillary vessels and atrial muscle in the human heart [18]. In skeletal muscle, there may be interfibrillar, perivascular, as well as intracellular fat deposition, as shown by quantitative histochemistry [9, 10].

From this assorted anatomical distribution of the adipocyte population there follows that the metabolic relation of FFA to glucose, *i.e.*, Randle cycle operation, depends on both systemic and local factors. For example, the rise in postprandial insulin concentrations restrains lipolysis in adipose ‘organs’ – via inhibition of hormone-sensitive lipase – but stimulates endothelial lipoprotein lipase, thereby facilitating dietary fat assimilation. Another example is tissue-specific sympathetic activation: an acute increase can quickly discharge FFA from epicardial fat into the myocardium, thereby imposing a further oxidative burden to an already ischemic area. Regular, intense exercise, on the other hand, repletes intramyocellular lipids while reducing VAT mass [19–21]. Chronic adrenergic overdrive to the kidney, which is associated with refractory hypertension, likely flushes FFA from perirenal AT to cortical nephrons, with consequences that may depend on the degree of renal dysfunction and the concomitance of other risk factors [22]. Atherosclerotic plaques harbor triglyceride-rich particles, the turnover of which is poorly understood [23].

These and other local effects, which would hardly be detected as changes in circulating FFA levels, are still incompletely characterized and are worthy of further investigation, especially when of potential clinical relevance.

## Cellular phenotype

Not all adipose tissues are alike [8, 24–27]. While adipocytes generally are metabolically very active (particularly when considering that their cytoplasm is only ~ 10% of the cell volume), VAT cells have a faster FFA-glucose turnover than SAT counterparts [28, 29]. With weight gain, human gluteofemoral SAT mass expands also through an increase in adipocyte number, thereby allowing for long-term nutrient storage, while VAT depots expand predominantly by an increase in fat cell size [8]. Furthermore, gluteofemoral fat contributes to ~ 25% of basal lipolysis [30] and is more sensitive to the antilipolytic effect of insulin than abdominal SAT [31]. Also, SAT seems to have a higher uptake of FFA and VLDL-triglycerides than chylomicron-triglycerides, indicating a preferential accumulation of recycled rather than dietary fat. Gluteofemoral SAT is believed to exert a protective role against metabolic and cardiovascular diseases [32]. In addition to protecting against ectopic fat deposition, due to a differential regulation of fatty acid release and uptake at the level of the adipocyte, gluteofemoral SAT secretes more beneficial adipokines and less proinflammatory molecules compared to abdominal fat [32].

On the trunk region, Scarpa’s fascia separates two SAT compartments, superficial and deep (Fig. 1). It has been observed that the superficial SAT layer is only weakly associated with insulin resistance, whereas the deep abdominal pad shows a robust inverse relation to insulin action [11]. This suggests that the cellular phenotype of the ‘deep’ SAT is intermediate between the ‘superficial’ SAT and VAT. VAT, on the other hand, includes omental and mesenteric depots, and represents an important risk factor for type 2 diabetes (T2D) and cardiovascular diseases. In particular, omental fat has an elevated lipolytic activity [31], and becomes resistant to the antilipolytic effect of insulin as it hypertrophies [33]. Hypertrophic abdominal VAT releases inflammatory cytokines [34], and is infiltrated by macrophages and T-cells, which in turn secrete inflammatory cytokines [35, 36].

Both visceral and subcutaneous adipocytes exhibit more marked histologic abnormalities in T2D than non-diabetic (ND) obese subjects [9]. In morbidly obese patients, adipocytes are enlarged in both subcutaneous and visceral depots, with evidence of cell stress, degeneration and necrosis. An enlarged adipocyte size is associated with increased lipolysis and depressed insulin action [9]. Hypertrophic adipocytes release less adiponectin and more inflammatory cytokines [37]. When adipocytes reach a critical size, macrophages infiltrating the adipose tissue surround dead cells forming ‘crown-like’ structures. The “critical death size” is lower in VAT adipocytes, thus macrophage infiltration and crown-like structures are more abundant in VAT than SAT, more prevalent in diabetic than nondiabetic obese subjects, and are related to insulin resistance [9]. Substantial weight loss – as occurs with bariatric surgery – is associated with improvements of both metabolic and morphological alterations [9, 38]. In parallel with changes in AT, skeletal muscle insulin sensitivity improves and intramuscular lipid is flushed out, while interfibrillar and perivascular fat depots are less affected [9, 10].

Another related consequence of excessive lipid loading is the build-up of bioactive lipid metabolites (such as ceramides, diacylglycerols, and long-chain fatty acyl-CoAs), which worsen cellular dysfunction and insulin resistance [39]. The improvement in insulin sensitivity that follows bariatric surgery is accompanied by an abatement in plasma proinflammatory biomarkers [9] and a reduction in liver steatosis [40, 41].

A special visceral adipose depot is the epicardial fat (EF). Epicardial adipocytes are smaller than adipocytes in other SAT or VAT depots, probably due to the greater number of preadipocytes compared to mature adipocytes. In addition to providing mechanical protection, EF represents a readily accessible energy source because its high FFA turnover allows direct diffusion of FFA to the adjacent myocardium [42]. However, hypertrophic EF – as in obesity with or without T2D –, is associated with increased cardiac work leading to cardiac hypertrophy, atrial enlargement, diastolic dysfunction, and coronary artery disease [43, 44]. Additionally, EF secretes proinflammatory adipokines, which are transported into the adjacent myocardium through vasocrine and/or paracrine pathways with further detrimental effects on heart function [43].

Additionally, an expanded EF may provide a chronic FFA overload to the heart, with subsequent intramyocardial fat accumulation. Such myocardial steatosis has been observed in subjects with obesity, diabetes, and metabolic syndrome, and may contribute to the development of the cardiac dysfunctions. Myocardial steatosis has also been reported in subjects with coronary artery disease [45], suggesting that myocardial hypoxia may itself have a role in myocardial fat accumulation. Histochemical analysis of atrial myocardium specimens of patients with or without coronary artery disease shows that fat infiltration inside cardiomyocytes is quite frequent, whereas adipocyte infiltration of the myocardium is not [18].

## FFA and glucose: metabolic factors

At the cellular level, the contribution of AT to glucose metabolism can be described as the balance between influx and outflow of substrates and hormones. In the fasting state, triglycerides packaged in cytoplasmic droplets are continually hydrolyzed to FFA and glycerol (by hormone-sensitive lipase, HSL), while the glycerophosphate generated via glycolysis provides the backbone for FFA reesterification. In the fasting state, the net adipocyte FFA balance is negative, so that FFA are released into the bloodstream, where they build up the concentration (~ 0.5 mM for FFA and 70–90 µM for glycerol) to which other organs (muscle, liver, heart, kidney, brain) are exposed. Under these conditions, the determinants of tissue FFA uptake are plasma FFA concentration, blood flow rate, and individual tissue fractional FFA uptake. Upon feeding, the surge in circulating insulin very efficiently restrains adipocyte lipolysis while stimulating glucose uptake; higher glycerophosphate generation from glucose helps suppressing lipolysis and stocking up triglycerides. Under these conditions, circulating FFA levels fall drastically, consequently tissue FFA uptake is reduced, and glucose uptake and utilization are augmented in proportion to individual tissue insulin sensitivity. This alternating switch of substrate supply, which has been termed metabolic flexibility [46], ensures appropriate energy loading and mobilization in phase with the feeding cycle.

The principal mechanism linking AT accumulation and insulin resistance is based on the glucose-fatty acid cycle or Randle cycle. In their experiments, Randle and colleagues [47] showed that adding fatty acids to perfused rat diaphragms and heart muscle increased fatty acid oxidation at the expense of carbohydrate oxidation. The increased FFA oxidation led to higher levels of intracellular acetyl-CoA and citrate, with a consequent inhibition of pyruvate dehydrogenase and phosphofructokinase. The resulting accumulation of glucose-6-phosphate caused inhibition of glucose phosphorylation and glucose uptake. Later works by Shulman et al. [48, 49, 50] added inhibition of glucose transport as a further mechanism of inhibition of glucose utilization.

Randle cycle is not the only mechanism implicated in the insulin resistance of obesity. In fact, chronic low-grade inflammation in adipose tissue may contribute to the development of insulin resistance and T2D [51]. Adipose tissue secretes proinflammatory cytokines, such as TNFalfa and others, which activate signals involved in inhibiting insulin action [52]. An increased lipolytic activity can also directly affect insulin signalling through the activation of serine kinases via fatty acid metabolites [53]. Furthermore, hypertrophic adipose depots are infiltrated by macrophages and T-cells which also express high levels of inflammatory cytokines [35, 54]. Macrophage infiltration is reported to coincide with the appearance of insulin resistance [55].

## Role of adipose tissue blood flow

**Fig. 3:**
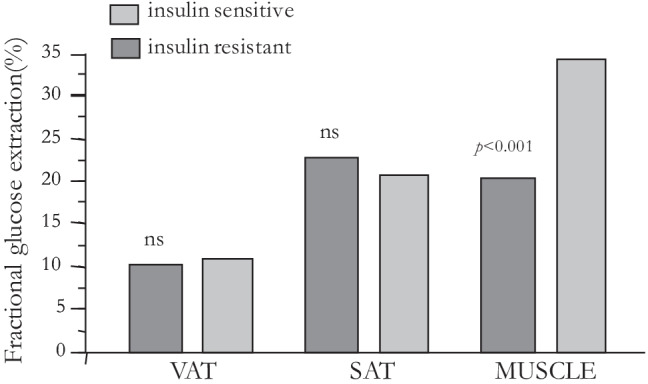
Fractional glucose uptake by visceral (peritoneal) adipose tissue (VAT), femoral subcutaneous adipose tissue (SAT), and femoral skeletal muscle in insulin sensitive and insulin resistant individuals. Modified from Ferrannini et al. [62]

Like any other tissue, AT metabolic activities, and FFA turnover in particular, are influenced by blood supply [56, 57]. Fat is highly vascularized, and variations in blood flow facilitate storage as well as removal of lipids. Human studies have demonstrated that in obese subjects AT shows reduced capillarization and cellularity [58–61]. Also, impaired regulation of adipose tissue blood flow (ATBF) has been linked with obesity and insulin resistance [12, 57]. In recent human studies combining positron-emitting tomography (PET), bioelectrical impedance, magnetic resonance imaging, and fat biopsies to simultaneously measure fat mass, adipocyte volume and number, blood flow, and FFA uptake in the fasting state [61], it was found that: (a) in lean subjects, tissue-specific (*i.e.,* per unit mass) blood flow and FFA uptake were higher in VAT than in SAT or skeletal muscle, in line with the higher metabolic rate of VAT; (b) in obese, nondiabetic individuals, adipocytes cell volume was lower in VAT than SAT; (c) in the obese group cellularity per unit mass was reduced in SAT, and tissue-specific blood flow to both SAT and VAT was reduced, as compared to lean subjects; (d) in obese individuals, total FFA uptake into SAT and VAT was increased *vs* lean subjects; however, when expressed per unit of tissue mass, neither measure differed between obese and lean. Collectively, these data confirm that VAT is hyperperfused and metabolically overactive as compared to SAT, that SAT is hypocellular in the obese, and that the higher FFA uptake of the obese is driven mostly by the expanded tissue mass. A subsequent study [62] – using PET to measure both blood flow and glucose uptake during a euglycemic hyperinsulinemic clamp – confirmed that tissue-specific blood flow and glucose uptake were higher in intraperitoneal VAT than femoral SAT. When glucose uptake was normalized by the corresponding blood flow, *i.e.,* calculating fractional glucose extraction, SAT was more avid than VAT but still 50% less avid than resting femoral muscle. Furthermore, at either fat site there was no difference between insulin sensitive and insulin resistant subjects, whereas in skeletal muscle fractional glucose extraction was ~ 40% reduced in insulin resistant *vs* insulin sensitive individuals (Fig. 3). Thus, reduced blood supply is an important factor for the impairment of *in vivo* insulin-mediated glucose uptake in both subcutaneous and visceral fat. In contrast, the insulin resistance of glucose uptake in resting skeletal muscle is predominantly a cellular defect.

## Role of adipose tissue oxygenation

Mature adipocytes require large amounts of ATP to maintain their activities (lipolysis, fatty acid β-oxidation, and fatty acid synthesis); ATP is generated in mitochondria from FFA ß-oxidation and in the tricarboxylic acid cycle [63], therefore mitochondria play a pivotal role in AT. Mitochondria can be dysfunctional in several organs; important contributor to mitochondrial dysfunction is the overload of nutrients characteristic of obesity [64–66]. Reduced mitochondrial function has also been implicated in the etiology of T2D [67]. In a study of morbidly obese subjects, we found that adipocytes contained rare mitochondria, and weight loss by bariatric surgery was associated with an increase of mitochondria number and size in parallel with a reduction in adipocyte size and an improvement in insulin sensitivity [9]. Another plausible mechanism connecting mitochondrial dysfunction to insulin resistance is the generation of reactive oxygen species (ROS) by mitochondria, which overwhelm cellular antioxidant capacity and lead to oxidative stress [68].This in turn leads to an accumulation of intracellular oxidized components, including lipids, proteins, nuclear and mitochondrial DNA, which are then released as damage-associated molecular patterns and constitute inflammatory triggers [69].

Some evidence suggests that differences in AT oxygenation contributes to the variability in insulin resistance. Studies conducted in rodent models have shown that the expansion of AT mass can cause AT hypoxia, leading to AT inflammation and fibrosis, and systemic insulin resistance [70]; increasing AT oxygen content, on the other hand, decreases AT inflammation and fibrosis and increases whole-body insulin sensitivity in obese mice [71]. Studies in humans find contrasting results, reporting that AT partial pressure of oxygen (pO2) was lower [59] or higher [72] in people with obesity than in lean individuals. In addition, the relationship between AT pO2 and whole body insulin sensitivity in humans is unclear because of conflicting data from different studies, reporting that AT pO2 was inversely associated [73] or not associated [74] with insulin sensitivity. A more recent study [75] evaluated subcutaneous SAT pO2, liver and whole-body insulin sensitivity, SAT expression of genes and pathways involved in inflammation and fibrosis, and systemic markers of inflammation in 3 groups of participants that were rigorously stratified by adiposity and insulin sensitivity into healthy lean, metabolically healthy obese, and metabolically unhealthy obese. The main finding was that SAT pO2 progressively declined from the lean to the metabolically healthy obese to the metabolically unhealthy obese group, and was positively associated with hepatic and whole-body insulin sensitivity. SAT pO2 was also negatively associated with AT gene expression of markers of inflammation and fibrosis.

Whether blood flow is important for AT oxygenation has not been clear. Studies in rodent models showed that the expansion of AT mass without adequate neovascularization can cause AT hypoxia, leading to AT inflammation and fibrosis, and systemic insulin resistance [70]. Recent human data on *in vivo* AT blood flow [62] and SAT oxygenation [75] do logically connect fat hypoperfusion and hypoxia as contributors of insulin resistance.

## Conclusions

Fat tissue is a master organ in metabolism, expressing its roles through diverse localization and contact with other tissues, blood flow dependency, changes in cellular phenotype, and biochemical flexibility. Its expandability, the highest among tissues, allows for long-term adaptation to overfeeding, by offering a sink for excess lipids as well as glucose. The cost of such adaptation is, however, significant: insulin resistance and its sequelae. The exact quantitative aspects, time course of development, and reversibility of fat hypoxia in human depots require further investigation.

## Abbreviations

AT: Adipose Tissue
ATBF: Adipose Tissue Blood Flow
ATP: Adenosine Triphosphate
EF: Epicardial Fat
FFA: Free Fatty Acids
HSL: Hormone-Sensitive Lipase
ND: Non-Diabetic
PET: Positron-Emitting Tomography
pO2: Partial Pressure of Oxygen
ROS: Reactive Oxygen Species
SAT: Subcutaneous Adipose Tissue
T2D: Type 2 Diabetic or Type 2 Diabetes
VAT: Visceral adipose Tissue
